# Orbital radiotherapy plus three-wall orbital decompression in a patient with rare ocular manifestations of thyroid eye disease: case report

**DOI:** 10.1186/s12902-018-0235-5

**Published:** 2018-02-06

**Authors:** Shuo Zhang, Yang Wang, Sisi Zhong, Xingtong Liu, Yazhuo Huang, Sijie Fang, Ai Zhuang, Yinwei Li, Jing Sun, Huifang Zhou, Xianqun Fan

**Affiliations:** Department of Ophthalmology, Ninth People’s Hospital, Shanghai Jiao Tong University School of Medicine, No. 639 ZhiZaoJu Road, Shanghai, 200011 China

**Keywords:** Thyroid eye disease, Dysthyroid optic neuropathy, Orbital radiotherapy, Three-wall decompression

## Abstract

**Background:**

Thyroid eye disease (TED) is a debilitating autoimmune orbital disease that is often a result of Graves’ disease. Dysthyroid optic neuropathy (DON) is a rare but sight-threatening manifestation of TED with therapeutic challenges that can potentially lead to visual loss.

**Case presentation:**

A 74-year-old man experienced active TED with extremely severe redness and swelling of the conjunctiva, loss of visual acuity and exacerbation of disfiguring proptosis. Computed tomography revealed the involvement of extraocular muscles resulting in optic nerve compression. He was in poor general condition and was intolerant to steroids. To achieve the optimal operating conditions for orbital decompression surgery, the patient was initially treated with orbital radiotherapy. The patient responded well, with improvements in clinical activity score and visual acuity.

**Conclusion:**

This case demonstrates a rare and severe case of DON with therapeutic challenges. To date, no cases has been reported of a patient with such severe and unusual ocular manifestations. Early awareness of the occurrence of optic nerve compression and prompt treatment are important to prevent irreversible outcomes. Orbital radiotherapy should be considered as a useful surgery-delaying alternative for DON, especially in patients who have contraindications to steroids.

## Background

Thyroid eye disease (TED) is an autoimmune orbital disease characterized by inflammation and edema of the periorbital connective tissues that results in expansion of the extraocular muscles and fat in the orbit [1]. It typically affects patients with hyperthyroidism due to Graves’ disease.

Dysthyroid optic neuropathy (DON) is a rare but potentially sight-threatening complication that occurs in patients with TED. Only 3–5% of patients with TED suffer from DON [2]. High doses of intravenous methylprednisolone (iv-MP) therapy are usually the first-line treatment. Orbital decompression surgery should be performed for patients who fail to adequately respond to iv-MP [3]. The management of patients with DON with contraindications to steroids and urgent surgery remains uncertain. Orbital radiation is administered to patients without DON in most cases [3]. However, limited reports have described the efficacy of orbital radiotherapy and subsequent decompression surgery in DON patients.

Herein, we present a rare and severe case of active DON treated with orbital radiotherapy followed by three-wall orbital decompression in a poor surgical candidate previously intolerant to steroids. To the best of our knowledge, a case of active DON with such severe and unusual ocular manifestations has not been previously reported. This case has major clinical significance because it highlights the potential severe presentation of DON and suggests that orbital radiotherapy can be an optimal surgery-delaying treatment for patients who have contraindications to steroids.

## Case presentation

A 74-year-old man presented to the Department of Ophthalmology, Shanghai Ninth People’s Hospital, for extremely severe redness and swelling of the conjunctiva, loss of visual acuity and exacerbation of disfiguring proptosis. The patient had a history of Graves’ disease and underwent a bilateral subtotal thyroidectomy in 1978. He experienced relapse of Graves’ disease three months after surgery and was treated with methimazole with limited response. Due to uncontrolled hyperthyroidism, the patient underwent a left total thyroidectomy in 1985 and radioiodine treatment in 2002. He exhibited proptosis 20 years ago and the proptosis had gradually worsened over the previous years. He underwent iv-MP therapy with doses of 0.84 g (consecutive daily infusions of 0.12 g for 3 days and 0.08 g for 6 days) in 2014 and with doses of 1.8 g (consecutive daily infusions of 0.48 g for 3 days, 0.24 g for 1 day and 0.12 g for 1 day) twice in 2015 with limited response. The patient also suffered from intolerable side effects of steroids, such as dizziness and headache, had profound muscle weakness and was unable to walk. He also experienced iv-MP-related hepatotoxicity with elevated alanine aminotransferase (ALT) levels. The patient had a history of myocardial infarction, atrial fibrillation and multiple lacunar infarctions. He denied any other comorbid conditions such as hypertension and diabetes mellitus.

**Fig. 1:**
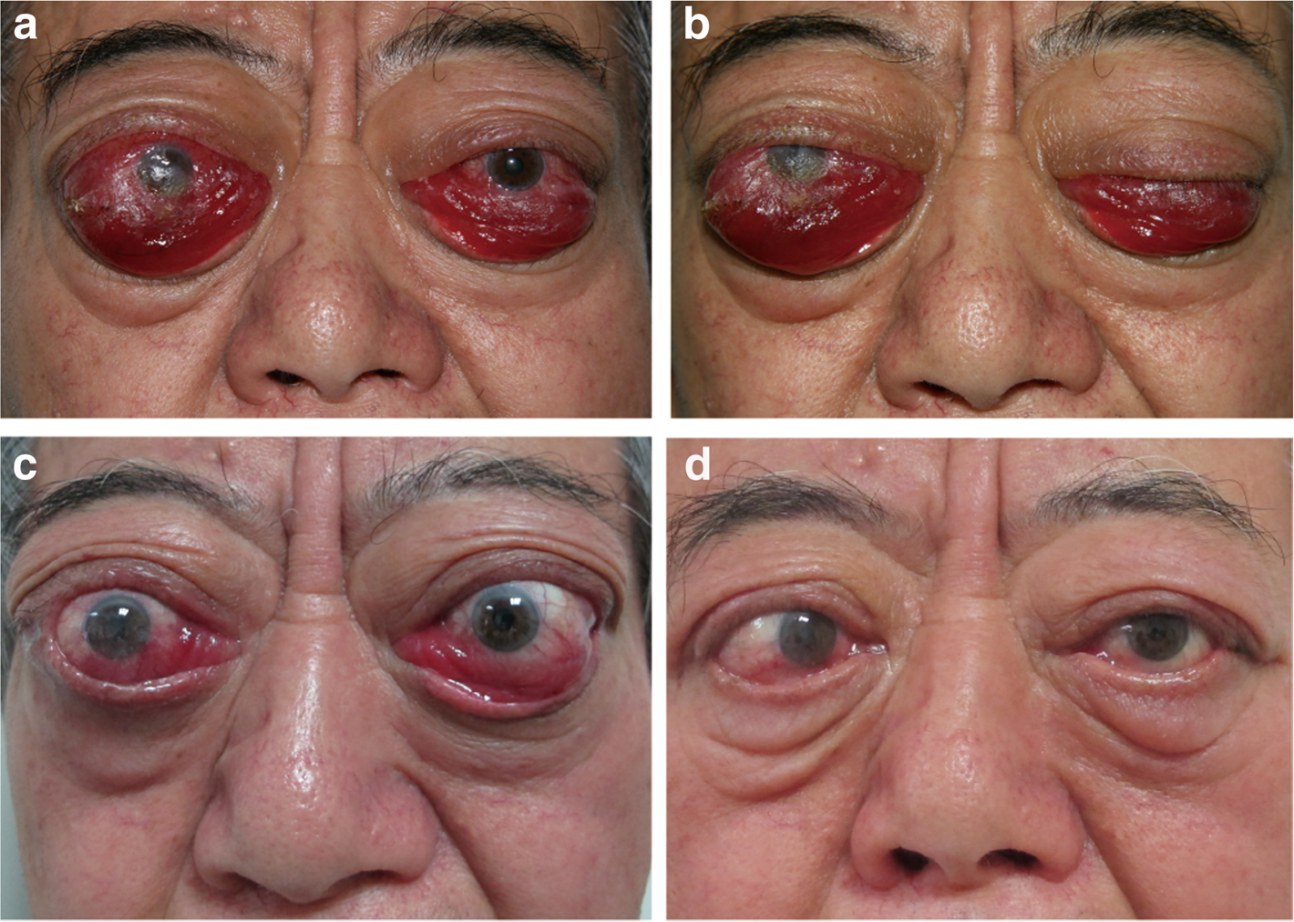
Ophthalmologic symptoms of the patient. A 74-year-old man developed highly active TED with DON. An ophthalmic examination showed eyelids edema, redness of the conjunctiva, chemosis and severe conjunctival prolapse (**a** and **b**). One month after orbital radiotherapy (**c**). Six months after bilateral orbital decompression (**d**)

**Fig. 2:**
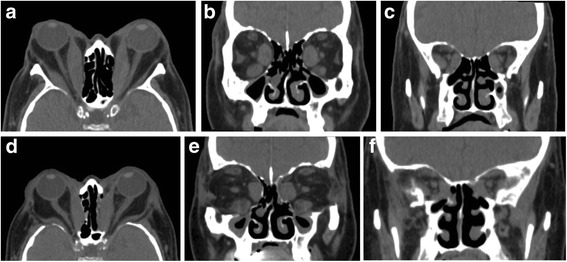
Preoperative and postoperative computed tomography (CT) of the orbit. Preoperative axial (**a**) and coronal (**b** and **c**) CT images showing proptosis, enlargement of extraocular muscles and apical crowding. Postoperative axial (**d**) and coronal (**e** and **f**) CT images showing the reduction in proptosis and relief of apical crowding

An ophthalmic examination showed reduction of best corrected visual acuity to hand movement in the right eye and 0.3 in the left eye, eyelids edema, swelling and hyperemia of the caruncle and plica, redness of the conjunctiva, remarkable chemosis and severe conjunctival prolapse (Fig. 1a and b). He was found to have severe restrictions in ocular movement in all directions with pain on attempted upward and downward gaze and spontaneous retrobulbar pain. He suffered from lagophthalmos and severe corneal exposure with diffuse punctate keratopathy and ulcers. Color vision, pupillary function and optic discs were normal in both eyes. His clinical activity score (CAS) was 6 points. Exophthalmometry showed proptosis of 32 mm of the right eye and 30 mm of the left eye. His intraocular pressures were 11 mmHg and 12 mmHg for the right and left eyes, respectively. He exhibited a free triiodothyronine (FT3) level of 3.43 pg/mL, low free thyroxin (FT4) level of 0.54 ng/dL, low thyroid-stimulating hormone (TSH) level of 0.06 μIU/mL, thyroid stimulating hormone receptor antibody (TRAb) level greater than 40.00 IU/L, an extremely high thyroid peroxidase antibody (TPOAb) level of 511.50 IU/mL, an elevated thyroglobulin antibody (TgAb) level of 433.10 IU/mL, high C-reactive protein (CRP) level of 2.06 mg/dL, high interleukin-6 (IL-6) level of 127.58 pg/mL, high tumor necrosis factor-α (TNF-α) level of 259.42 pg/mL, normal immunoglobulin (Ig) G, IgA, IgM and IgG4 levels and an elevated IgE level of 465 IU/mL (Table 1). The patient received 7.5 mg of methimazole per day and 12.5 μg of levothyroxine sodium per day. An orbital computed tomography (CT) scan showed enlargement of the extraocular muscles bilaterally with marked enlargement of the medial rectus and inferior rectus muscles resulting in apical crowding and optic nerve compression (Fig. 2a–c). A diagnosis of active TED with DON and exposure keratitis was made [3].

**Table 1:** Laboratory data before and after radiotherapy

	Before radiotherapy	Two months after radiotherapy	Normal range
FT3	3.43	3.48	2.5–3.9 pg/mL
FT4	0.54	0.45	0.58–1.64 ng/dL
TSH	0.06	1.99	0.34–5.6 μIU/mL
TPOAb	511.5	603.6	0–9 IU/mL
TRAb	> 40.00	> 40	0–1.75 IU/L
TgAb	433.1	122.9	0–115 IU/mL
CRP	2.06	0.60	0–0.80 mg/dL
IL-6	127.58	42.40	< 3.4 pg/mL
TNF-α	259.42	86.33	< 8.1 pg/mL
IgG	9.56	11.20	7–16 g/L
IgA	2.23	2.23	0.7–4 g/L
IgM	0.54	0.55	0.4–2.3 g/L
IgE	465.0	312.0	0–100 IU/mL
IgG4	0.555	0.637	0.03–2.01 g/L

*Abbreviations*: *FT3* free triiodothyronine, *FT4* free thyroxin, *TSH* thyroid-stimulating hormone, *TRAb* thyroid stimulating hormone receptor antibody, *TPOAb* thyroid peroxidase antibody, *TgAb* thyroglobulin antibody, *Ig* immunoglobulin, *CRP* C-reactive protein, *IL-6* interleukin-6, *TNF-α* tumor necrosis factor-α

**Fig. 3:**
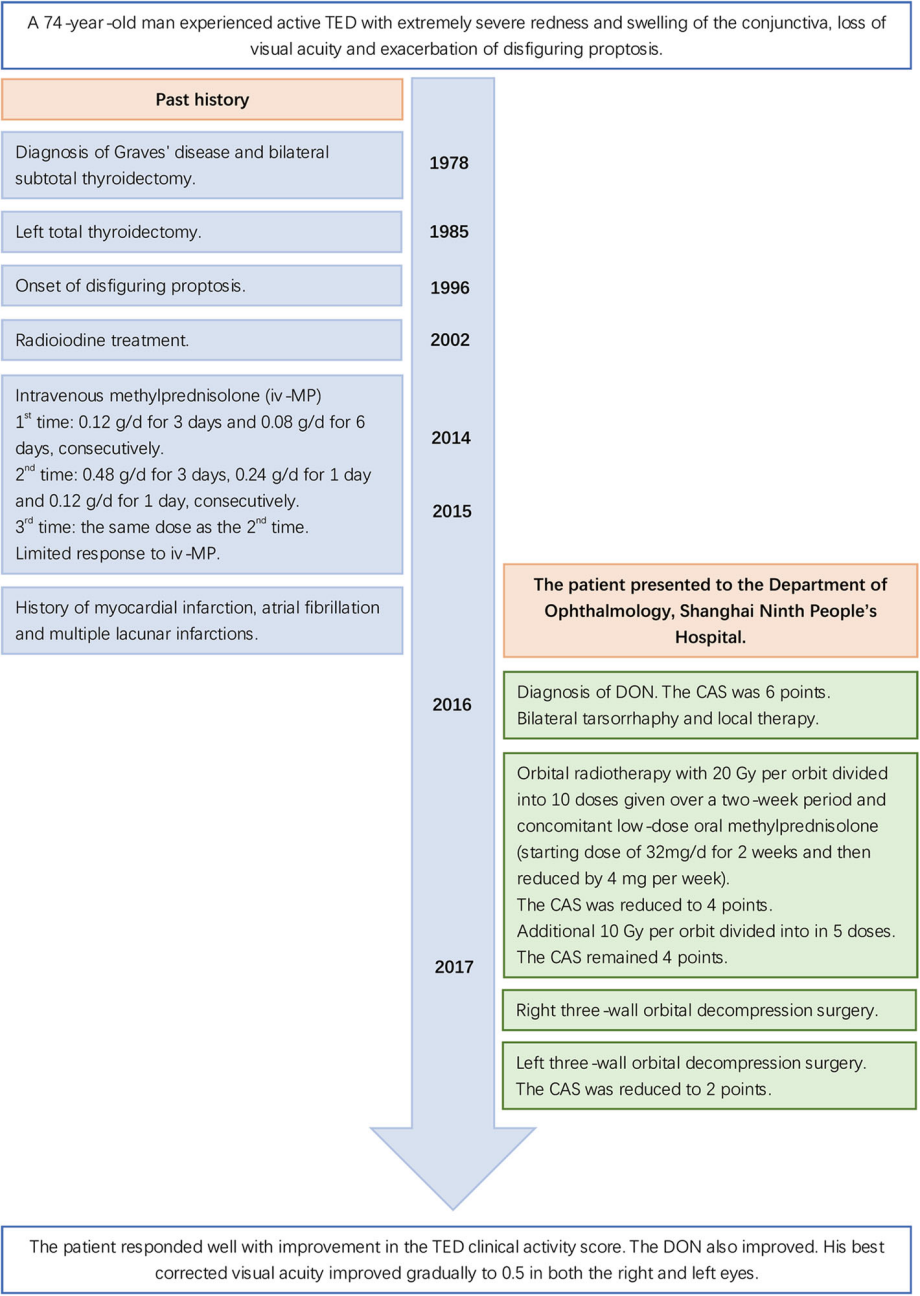
Timeline of interventions and outcomes

The patient was evaluated as an urgent case and required prompt intervention (Fig. 3). Bilateral tarsorrhaphy and local therapy were performed to prevent worsening of chemosis and development of corneal ulcers. The patient was suggested to start 500 mg iv-MP for 3 consecutive days, but he had strong concerns about the use of glucocorticoids. At that time, the severe and terrible ocular signs including remarkable chemosis and severe conjunctival prolapse prevented immediate decompression surgery due to high intraorbital pressure and inaccessibility to the small surgical field. Therefore, decompression surgery was deemed high risk for optic nerve impairment. His poor general status due to multiple comorbidities and increased risk under anesthesia also interfered with urgent decompression surgery. Therefore, orbital radiotherapy with 20 Gy per orbit divided into 10 doses was given over a two-week period. Concomitant low-dose oral glucocorticoids were administered. The chemosis, conjunctival prolapse and corneal lesions significantly improved within 1 month (Fig. 1c). Spontaneous retrobulbar pain and pain on attempted eye movement were also significantly decreased. His CAS was also reduced by 2 points. Thus, an additional 10 Gy per orbit divided into in 5 doses was given. However, the impairment in best corrected visual acuity remained. Orbital radiotherapy significantly improved the ocular signs and offered the possibility of decompression surgery.

Two months later, after extensive discussion of the risks and benefits of surgery, such as haemorrhage, cerebrospinal fluid (CSF) leakage, postoperative diplopia, postoperative lateral orbit depression and scarring, the patient agreed to proceed with right three-wall orbital decompression surgery under general anesthesia. A horizontal skin incision along with the double eyelid fold and lateral to the lateral canthus was made for lateral wall decompression. The deep lateral wall into the trigone of the greater wing of the sphenoid was removed, and the lateral orbital rim was removed en bloc. Transconjunctival and transcaruncular incisions were made for medial and inferior wall decompression. The patient subsequently underwent left three-wall orbital decompression using the same surgical techniques (Fig. 1d).

Following bilateral orbital decompression, the patient’s best corrected visual acuity gradually improved. Six months following surgery, the final best corrected visual acuity improved to 0.5 in both the right and left eyes. The patients exhibited improvement in the eyelids edema and swelling and hyperemia of the caruncle and plica. His CAS was reduced to 2 points. Postoperative CT scans demonstrated relief of bilateral crowding in the orbital apex (Fig. 2d–f).

## Discussion and conclusion

DON is a rare and severe complication of TED that can lead to definite visual loss [3]. Compression of the optic nerve at the orbital apex due to extraocular muscles enlargement and inflammatory reaction are the main causes of DON [2]. The diagnosis of DON is made based on the presence of a decreased visual function due to optic neuropathy secondary to TED [3, 4]. The European Group on Graves’ Orbitopathy (EUGOGO) reported a prospective case series of 47 patients to identify clinical manifestations of DON [5]. The EUGOGO study found that patients with DON may not have severe proptosis and orbital inflammation. Evidence of optic nerve compression on imaging was one of the most sensitive clinical features, consistent with the findings in our patient. In our patient, the proptosis was 32 mm in the right eye and 30 mm in the left eye, which is far greater than the mean proptosis of 22.1 mm in both eyes reported in the EUGOGO study. To the authors’ knowledge, this is the first case of a patient with such severe chemosis and conjunctival prolapse, which made the treatment more perplexing and challenging.

Glucocorticoids, orbital radiotherapy, orbital decompression, immunosuppressive therapy and biological drugs are available for the management of TED [3]. Iv-MP and prompt orbital decompression, if necessary, are is still considered to be the standard treatment for DON [3]. A randomized trial including 15 patients with active TED and DON suggested that immediate surgery does not result in better outcomes, and systemic glucocorticoids appeared to be the optimal first-line treatment [6]. To the best of our knowledge, no reports are available regarding the treatment of patients with DON with contraindications to steroids and surgical intolerance. Orbital radiotherapy plays an important role in controlling the inflammatory process of TED by inducing apoptosis or disrupting the functions of B and T lymphocytes, macrophages, or orbital fibroblasts and therefore reducing the secretion of proinflammatory cytokines from activated lymphocytes [7, 8]. A total dose of 20 Gy is commonly used [9, 10]. Grassi et al. reported significant early reduction in CAS and ocular motility disturbances after orbital radiotherapy in patients without DON [8]. Another study demonstrated that all patients with TED showed regression of the disease with combined iv-MP and orbital radiotherapy or iv-MP therapy alone. Two of fifty-nine patients undergoing iv-MP therapy developed DON during the follow-up period, but no patients receiving combined iv-MP and orbital radiotherapy developed DON [11]. In addition, some studies have indicated that the high incidence of IgE elevation in Graves’ disease suggested a difference in the autoimmune processes of the disease with and without IgE elevation [12, 13]. We found elevated serum IgE levels in our case, suggesting that IgE may also participate in the immunopathogenesis of TED. The patient’s IgE level decreased after orbital radiotherapy. The existing literature provides evidence of the efficacy of radiotherapy and its protective role against TED. Therefore, we advocate orbital radiation as the ideal therapy in patients with DON who have contraindications to steroids and cannot tolerate surgery, especially those with elevated IgE levels.

The reported effects of radiotherapy on visual acuity have been variable [8, 14]. In our case, no significant improvement in visual acuity was found after radiotherapy. Since the recovery of visual acuity is the main goal of treatment in patients with DON, additional orbital decompression is often required. Decompression is the only definitive treatment to relieve apical crowding, save the vision and reduce considerable exophthalmos [3]. Medial and inferior wall decompression led to marked improvement in visual acuity [15]. Balanced medial and lateral wall decompression has shown equal efficacy in terms of saving vision and lowering the rate of postoperative diplopia [16]. Kikkawa et al. proposed graded orbital decompression based on the severity of exophthalmometry, and a mean proptosis reduction of 8.9 ± 3.4 mm was obtained in the three-wall decompression group [17]. In the present case, a proptosis reduction of 10 mm in the right eye and 7 mm in the left eye was achieved. Orbital radiotherapy ameliorated the inflammatory reactions in the orbit and offered time to prepare for orbital decompression surgery.

Preoperative radiotherapy does not interfere with the outcomes of orbital decompression [18], and may prevent and control relapses in DON [19]. Shams et al. reported that the rate of DON was significantly reduced in patients receiving orbital radiotherapy in addition to corticosteroids in their study [19]. In our case, preoperative radiotherapy offered optimal operating conditions for orbital decompression surgery and eased the surgery.

In conclusion, DON is a rare disease in patients with TED. Apart from the well-known ocular manifestations of TED, severe conjunctival prolapse and apical crowding can occur, resulting in devastating sight loss. We suggest that orbital radiotherapy can be a temporizing treatment for patients with DON in poor general condition and with contraindications to steroids until the patients are well prepared for orbital decompression. Patients with elevated IgE levels are especially likely to benefit from radiotherapy. The efficacy of orbital radiation for DON has not been well investigated and further studies and clinical trials are needed.

## Abbreviations

CRP: C-reactive protein
DON: Dysthyroid optic neuropathy
FT3: Free triiodothyronine
FT4: Free thyroxin
Ig: Immunoglobulin
IL-6: Interleukin-6
TED: Thyroid eye disease
TgAb: Thyroglobulin antibody
TNF-α: Tumor necrosis factor-α
TPOAb: Thyroid peroxidase antibody
TRAb: Thyroid stimulating hormone receptor antibody
TSH: Thyroid-stimulating hormone
